# Supplementary material for: Modelling the erythroblastic island niche of dyserythropoietic anaemia type IV patients using induced pluripotent stem cells
Source: Front Cell Dev Biol. 2023 Apr 11;11:1148013. doi: 10.3389/fcell.2023.1148013 (PMC10126837; doi:10.3389/fcell.2023.1148013)
Supplement: Supplementary file 1 [file Table1.DOCX]

**Supplementary Tables:**

**Supplementary Table 1: Primers used for PCR and Sanger sequencing.**

| **Primer Name** | **Sequence** |
| --- | --- |
| KLF1 SDM NEB_FW | CGGGTCAGCTTGTCCGAGCGC |
| KLF1 SDM NEB_RV | CCACTACCGGAAACACACGGG |
| pZDonor_FW | CCGTCGACGCTCTCTAGAGCTAG |
| Int_RV | CCGTCGACGCTCTCTAGAGCTAG |
| 5’aavs1_FW | CGGAACTCTGCCCTCTAACGCTGCCG |
| 5’aavs1_RV | CTGCCAGATCTCTCGAGGCCCTGTGG |
| Ext_RV | ACAGCCCCAGGTGGAGAAACTG |
| KLF1 Sanger sequencing | CACTTTGTTCATCCTAGTCCCA |

**Supplementary Table 2: Primers used for qRT-PCR gene expression analyses**

| **Primer Name** | **Forward Primer Sequence** | **Reverse Primer Sequence** |
| --- | --- | --- |
| GYPA | ATATGCAGCCACTCCTAGAGCTC | CTGGTTCAGAGAAATGATGGGCA |
| TFRC | ATCGGTTGGTGCCACTGAATGG | ACAACAGTGGGCTGGCAGAAAC |
| SLC4A1 | CTGCTGGTGTTTGAGGAAGCCT | CACCAGCAGGATGAGCCAGAAG |
| ICAM4 | GGCACCCATTACACTGATGCTC | AGGCACTTGCATAGGTACGCAG |
| HBA1 | GACCCGGTCAACTTCAAGC | AGAAGCCAGGAACTTGTCCA |
| KLF1 WT | ACACCAAGAGCTCCCACCT | GTAGTGGCGGGTCAGCTC |
| KLF1 WT/E325K | ACACCAAGAGCTCCCACCT | GTAGTGGCGGGTCAGCT |
| IL33 | CCACCAAAAGGCCTTCACT | AAGGCAAAGCACTCCACAGT |
| IGFBP6 | CACAGGATGTGAACCGCAGAGA | CACTGAGTCCAGATGTCTACGG |

**Supplementary Table 3: Antibodies used for flow cytometry analyses**

| **Cell Surface Marker** | **Conjugated Fluorochrome** | **Manufacturer** | **Dilution** |
| --- | --- | --- | --- |
| CD43 | APC | 17-0439-42, Invitrogen | 1:100 |
| EpCAM | PE | 324205, Biolegend | 1:100 |
| CD235a | FITC | 349104, Biolegend | 1:200 |
| CD71 | APC | 17-0719-42, Invitrogen | 1:200 |
| CD45 | APC | 15577936, Ebioscience | 1:100 |
| CD93 | PE | 10804637, Ebioscience | 1:200 |
| 25F9 | E-FLUOR-660 | 15599866, Ebioscience | 1:20 |
| CD163 | PE-CY7 | 333614, Biolegend | 1:25 |
| CD169 | APC | 346007, Biolegend | 1:25 |
